# Structural and biochemical insights into human zinc finger protein AEBP2 reveals interactions with RBBP4

**DOI:** 10.1007/s13238-017-0483-6

**Published:** 2017-11-13

**Authors:** Aiai Sun, Fudong Li, Zhonghua Liu, Yiyang Jiang, Jiahai Zhang, Jihui Wu, Yunyu Shi

**Affiliations:** Hefei National Laboratory for Physical Sciences at Microscale and School of Life Sciences, University of Science and Technology of China, Hefei, 230027 China

**Dear Editor,**

Polycomb repressive complex 2 (PRC2) plays a critical role in organ development, adult homeostasis, and tumorigenesis via enzymatic activities to di- or tri-methylate lysine 27 of H3 (H3K27me2/me3), which is a hallmark of transcriptional repression and gene silencing (Margueron and Reinberg, 2011). The complex consists of the following four core components: EZH2/EZH1, SUZ12, EED, and RBBP4/RBBP7 (also known as RbAp48/RbAp46) in mammals. EZH2, EED, and SUZ12 are minimally required for PRC2 methylation catalysis (Cao and Zhang, 2004) and the complex structure with H3K27M has recently been reported (Justin et al., 2016). The crystal structure of Nurf55 (homolog of RBBP4 in *Drosophila*) in complex, with H3 or SUZ12, demonstrates that RBBP4 is essential for PRC2 to be able to interact with chromosomes (Schmitges et al., 2011). Moreover, the addition of RBBP4 results in an increase in PRC2 enzymatic activity (Cao and Zhang, 2004).

Beyond the core subunits, AEBP2 serves as an associating components of PRC2, which is deemed necessary for PRC2 recruitment and PRC2 activity modulation (Kim et al., 2009). Through interacting with RBBP4 and SUZ12, AEBP2 stabilizes the core PRC2 complex, thus significantly increases the HMTase activity of PRC2 (Cao and Zhang, 2004). Moreover, AEBP2 has been reported *in vivo* to locate to the known PcG target loci, which presents a bipartite architecture pattern, CTT(N) 15-23cagGCC (Kim et al., 2009).

All human isoforms of AEBP2 contain three Cys2His2 zinc fingers and an RRK-rich motif. Sequence alignments demonstrate that these regions are highly conserved in vertebrates (Figs. 1A and S1). To determine the domain architecture of AEBP2, we expressed the recombinant protein containing the three AEBP2 zinc fingers (Uniprot entry: Q6ZN18-1; residues Asn258–Gln357, hereafter referred to as “ZF1–3”). Additionally, we determined the solution structure of ZF1–3 using 3D NMR spectroscopy (Fig. 1, PDB ID: 5Y0U). The whole structure converges well, as the r.m.s.d. of the backbone and heavy atoms of the 20 lowest energy ensembles are 0.602 and 0.954, respectively (Table S1 and Fig. 1B). In addition, each of the zinc fingers converges well with a small r.m.s.d (Table S1).

**Figure 1:**
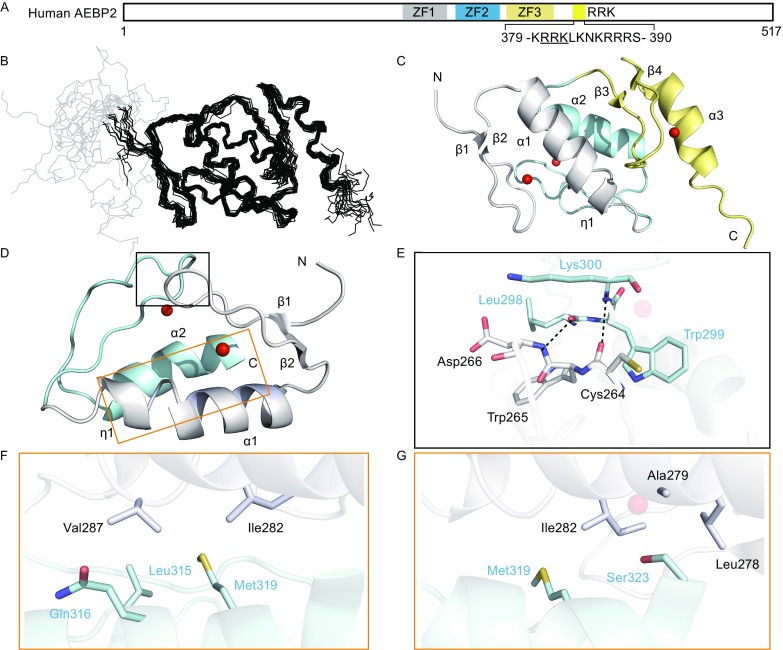
**All three zinc fingers of AEBP2 are composed of Cys2His2 zinc fingers**. (A) Schematic representation of the human AEBP2 protein, including the three zinc fingers and RRK rich motif. The first zinc finger is shown in gray bar, second in light blue bar and third in light yellow, respectively. The RRK rich motif is shown in yellow bar, with sequence shown as single letter amino acids. (B) Stereo image of the 20 lowest energy ZF1–3 backbone structures (PDB ID: 5Y0U). The overall structure is depicted, with the His_6_-tag shown in gray. (C) Cartoon representation of ZF1–3 highlights the secondary structural elements, color scheme as for (A), and spheres are zinc ions. (D) Overview of ZF1–2 packing as tCWCH2 domain. The inter-zinc finger interaction regions are squared with gray line and orange line, respectively. (E) Detail view of the loop region. The residues involved inter-zinc finger interactions are shown in sticks. Hydrogen bonds are delineated by black dashed lines. (F and G) Detail view of the helix region. The residues involved inter-zinc finger interactions are shown in sticks

The first zinc finger in AEBP2 (hereafter referred to as “ZF1”), containing residues Tyr261–His286, folds as a canonical Cys2His2 zinc finger architecture (Krishna et al., 2003), beginning with a well-defined anti-parallel β-sheet (sheet 1, β1–β2, residues Tyr261–Asn262 and Cys271–Phe272), followed by an α helix (α1, residues Ser275–Ile285). The residues Asp288–Gln290 after ZF1 fold as a small helix, η1 (Fig. 1C). In the second zinc finger domain (hereafter referred to as “ZF2”), excepting those with the α helix (α2, residues Gln312–Ser323), no β sheet was observed. The additional sequence Lys303–Tyr305 folds as a protruding outward loop (Fig. 1C). Similar to ZF1, the third zinc finger domain (hereafter referred to as “ZF3”) also forms a typical Cys2His2 zinc finger domain, with an α helix (α3, residues Gln342–His352) after a β-sheet (sheet 2, β3–β4, residues Phe328–Lys329 and Ser338–Phe339) (Fig. 1C).

Myriad interactions observed between ZF1 and ZF2 created the two zinc fingers combined as a tCWCH2 architecture (Hatayama and Aruga, 2010) (hereafter referred to “ZF1–2”; Fig. 1D). The van der Waals and hydrophobic interactions were observed between Cys264–Asp266 in ZF1 and Leu298–Lys300 in ZF2, contributing to inter zinc finger interactions. Moreover, the amide groups of Asp266 and Lys300 created hydrogen bonds with the carbonyl groups of L298 and Cys264, respectively (Fig. 1E). A hydrophobic core was observed between α1 in ZF1 and α2 in ZF2, which further contributed to the tCWCH2 architecture made by ZF1 and ZF2. Val287, coming after α1, was surrounded by Leu315, Gln316, and Met319 from α2, together with Met319 from α2 interacting with Ile282 from α1 (Fig. 2F). On the other end of the helix bundle, Ser 323 from α2 was surrounded by Leu278, Ala279 from α1 (Fig. 2G). All the interactions mentioned above can also be confirmed with the observed NOE restraints. A total of 56 inter-zinc finger cross NOE peaks are observed between ZF1 and ZF2, which are summarized in Table S2, with five representative columns presented in Fig. S2.

**Figure 2:**
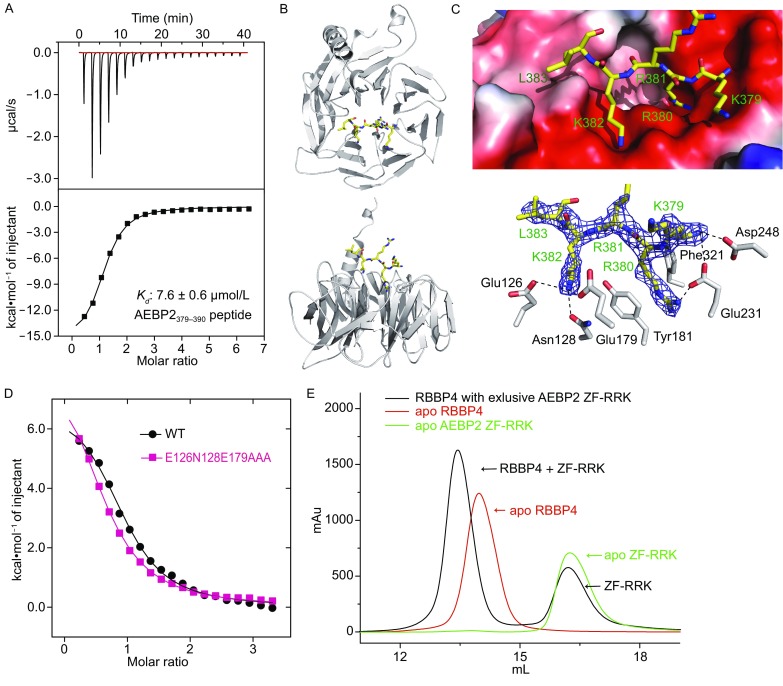
**Both the three zinc fingers and RRK rich motif interact with RBBP4**. (A) Determination of the affinity of AEBP2_379–390_ peptide for the RBBP4 protein using ITC. Data were fitted to a one-site binding model using Origin 7, and calculated binding parameters were ∆H = −15,890 ± 395 cal/mol, and ∆S = −29.9 cal/mol/deg. (B) Two orthogonal views of RBBP4 bound to the AEBP2_379–390_ peptide. The AEBP2_379–390_ peptide is shown in yellow, with RBBP4 in gray. (C) Recognition of the AEBP2_379–390_ peptide by RBBP4. Electrostatic surface potential representation of the binding pocket with the AEBP2_379–390_ peptide (shown in yellow stick model) (upward) and a simulated annealing omit map (blue) contoured at 1.0*σ* shows the electron density for the AEBP2_379–390_ peptide bound to RBBP4 (downward). The AEBP2_379–390_ peptide (labeled in green) is shown in yellow stick model, with RBBP4 residues (labeled in black) in gray stick model. Hydrogen bonds and salt bridge interactions are delineated by black dashed lines. (D) Determination of the affinity of AEBP2 ZF1–3 for RBBP4 of wild type and Glu126Ala/Asn128Ala/Glu179Ala mutation using ITC, respectively. Data were fitted to a one-site binding model using Origin 7. (E) The overlaid curves of apo RBBP4, apo AEBP2 ZF-RRK and mixture of RBBP4 with exccesive AEBP2 ZF-RRK by gel filtration assays on a same SuperdexTM 200 10/300 GL column

Cys2His2 zinc finger domains are known as the most abundant DNA-binding motifs (Razin et al., 2012). To validate whether AEBP2 ZF1–3 binds to double stranded DNA (dsDNA), we conducted fluorescence polarization assays with the 44-bp GC-rich, AT-rich and previously reported T1 dsDNA (Kim et al., 2009). Unlike the positive control tzap zinc finger protein (Li et al., 2017), which binds to all tested dsDNA, AEBP2 ZF1–3 does not interact with dsDNA under the same conditions tested (Fig. S3). AEBP2 ZF1–3 have also been tested with a G quadruplex sequence termed G3A4 reported previously bound to PRC2 complex (Wang et al., 2017) and also gets a negative results (Fig. S3). The feature ZF1–3 does not interact with tested DNA is likely due to the following reasons (Fig. S4). First, the residues located at positions −1, +2, +3 and +6 of AEBP2 ZF1 are Ser274, Pro276, Asp277 and Asp280, respectively, which are with negative charge. Second, with ZF1 and ZF2 combined as a tCWCH2 domain, although all potential DNA binding residues are exposed outside, AEBP2 ZF1–3 lacks the flexibility needed for DNA binding. Third, the linker between ZF1 and ZF2 was significantly longer than and distinct from the canonical TGE(K/R)P sequences found in the DNA-binding ZFs, which are critical parameters for their DNA-binding affinities (Laity et al., 2000). With longer linkers and insertion of extra sequence in ZF2, ZF1–3 may have functions other than DNA binding.

Previous research has shown that AEBP2 interacts with RBBP4 (Cao and Zhang, 2004). Via the sequence alignments, we found a conserved RRK-rich motif (residues Lys379–Ser390, hereafter referred to “AEBP2_379–390_”) near after the three zinc fingers in AEBP2 (Figs. 1A and S1). To validate whether the RRK-rich motif of AEBP2 can bind RBBP4, we conducted an ITC experiment using synthetic AEBP2_379–390_ peptide to titrate full-length RBBP4. RBBP4 binds to AEBP2_379–390_ peptide with a dissociation constant (*K*_d_) of 7.6 ± 0.6 μmol/L (Fig. 2A).

To provide structural insight into the recognition of AEBP2_379–390_ by RBBP4, we sought to solve the structure of the complex by X-ray crystallography. After crystallographic screening, we obtained the co-crystal of RBBP4 in the complex with the AEBP2_379–390_ peptide and determined its structure at a resolution of 2.15 Å (Table S3). Nearly identical to the previously determined structures, RBBP4 has a seven-bladed β propeller fold, with a protruding N-terminal α helix (Fig. 2B). In the complex structure, electron density corresponding to the first five residues (residues Lys379–Leu383) of AEBP2_379–390_ peptide could be traced.

The peptide lies on the axis channel of the smaller surface of RBBP4 (Fig. 2B). Arg380 inserts deeper into the axis channel of the smaller surface of RBBP4, with its side chain sandwiched by Tyr-181 and Phe-321 and with its guanidinium group creating electrostatic contact with Glu231. Lys382 binds to a well-defined cleft on the surface formed by blade 2 and 3 of RBBP4, with its ε-group specifically contacts with Glu126, Asn128, and Glu179. Moreover, Asn231 and Asn248 in RBBP4 create hydrogen bonds with the main chain amide group of Lys379.

To validate the crucial residues for conferring binding specificity, we conducted site-directed mutations in both RBBP4 and AEBP2_379–390_ peptide (Fig. S5A). ITC data demonstrated that binding affinities of AEBP2_379–390_ peptide to the RBBP4 with mutations Glu231Ala and Glu126Ala/Asn128Ala/Glu179Ala are reduced by 5- and 7-fold, respectively, compared with wild-type RBBP4 (Fig. S5A). In contrast to wild-type AEBP2_379–390_ peptide, the binding affinities of AEBP2_379–390_ peptide with mutations Arg380Ala/Lys382Ala to the RBBP4 were reduced by approximately 20-fold.

A similarly conserved R/K motif was found in FOG-1 (Lejon et al., 2011), PHF6 (Liu et al., 2015)and histone H3 (Schmitges et al., 2011) (Fig. S5B). Together with the two conserved arginines and lysines, there are always other residues assisting in the interactions, which provide a larger interface than AEBP2_379–390_ (Fig. S4C). For example, excepting Arg2 and Lys4, Ala7 and Lys9 of the N-terminal of histone H3 (residues 1–19) also contribute to the recognition to RBBP4. Given that H3 also participates in PRC2 regulation (Schmitges et al., 2011), whether there is a competition for RBBP4-binding between AEBP2 and H3 remains an open question requiring further inquiry.

The binding interface between AEBP2_379–390_ and RBBP4 is relatively small compared with PHF6, histone H3 and FOG-1, indicating that AEBP2_379–390_ may not have been the only region that participates in RBBP4 recognition. To validate whether the three AEBP2 zinc fingers interacts with RBBP4, we conducted an ITC experiment with AEBP2 ZF1–3 titrating to RBBP4. Of note, the interaction between these two proteins was confirmed with a dissociation constant (*K*_d_) of 10.1 ± 0.6 μmol/L (Fig. 2D). Furthermore, we expressed the recombinant protein containing both ZF1–3 and the RRK-rich motif (residues Asn258–Asp396, hereafter referred to “ZF-RRK”). During gel filtration assays, a complex peak of RBBP4 bound to AEBP2 ZF-RRK was confirmed with overlaid curves of apo RBBP4 and apo AEBP2 ZF-RRK, together with corresponding SDS-PAGE (Figs. 2E and S6). The mixture peak apex of RBBP4 bound to AEBP2 ZF-RRK is at 13.44 mL, with apo RBBP4 at 13.96 mL. The apo AEBP2 ZF-RRK peak is a little asymmetric and the peak apex is at 16.26 mL, which lags little behind the exclusive AEBP2 ZF-RRK (at 16.21 mL), which may be due to degradation.

To test whether ZF1–3 and RRK-rich motif bind to the distinct regions of RBBP4, we conducted ITC assays using AEBP2 ZF1–3 to titrate RBBP4 with Glu126Ala/Asn128Ala/Glu179Ala mutation. AEBP2 ZF1–3 binds to RBBP4 Glu126Ala/Asn128Ala/Glu179Ala mutation with a dissociation constants (*K*_d_) of 16.0 ± 0.4μmol/L, which is about 1.5 fold compared with the wild type (Fig. 2D), indicating that ZF1–3 and RRK-rich motif bind distinct regions on RBBP4.

In summary, we have demonstrated that there are multiple inter-zinc finger interactions between ZF1 and ZF2, indicating that AEBP2 ZF1–3 may not bind to dsDNA. This finding was further confirmed through fluorescence polarization assays. In this work, we found the molecular basis of AEBP2 RRK-rich motif interacting with RBBP4, which shares the same binding interface with histone H3 and is relatively smaller. More importantly, to our knowledge, we are the first to identify AEBP2 ZF1–3 and RRK-rich motif as dual recognition for RBBP4. Our data demonstrate the structural basis and functional significance of AEBP2 associated with PRC2 activity. Further identification and analysis of the molecular basis between AEBP2 ZF-RRK and RBBP4 would provide valuable new insights into PRC2 activity regulation.

## Electronic supplementary material

Below is the link to the electronic supplementary material.

Supplementary material 1 (PDF 1069 kb)
