# Supplementary material for: Structural and biochemical insights into human zinc finger protein AEBP2 reveals interactions with RBBP4
Source: Protein Cell. 2017 Nov 13;9(8):738–42. doi: 10.1007/s13238-017-0483-6 (PMC6053351; doi:10.1007/s13238-017-0483-6)
Supplement: Supplementary file 1 — Supplementary material 1 (PDF 1069 kb) [file 13238_2017_483_MOESM1_ESM.pdf]

Structural and Biochemical Insights into Human Zinc Finger Protein AEBP2 Reveals Interactions with RBBP4

KEY WORDS: AEBP2, Cys2His2 zinc finger, RBBP4, PRC2.

Aiai Sun[1], Fudong Li[1], Zhonghua Liu[1], Yiyang Jiang[1], Jiahai Zhang[1], Jihui Wu[1]*, Yunyu Shi[1]*
[1] Hefei National Laboratory for Physical Sciences at Microscale and School of Life Sciences, University of Science and Technology of China, Hefei, Anhui 230027, China
* Correspondence: yyshi@ustc.edu.cn (Yunyu Shi); wujihui@ustc.edu.cn (Jihui Wu).

SUPPLEMENTARY FIGUREs

Figure S1. Sequence alignment of orthologs of AEBP2 (Residues Asn258 - Asp396) protein from diverse vertebrate species. The cysteines and histidines coordinating zinc ions are marked with stars. The residues corresponding to DNA binding zinc fingers involved in DNA interaction are marked with triangles. The RRK rich motif are highlighted with yellow background, with crucial residues contributing to RBBP4 recognition marked with red circles.

Figure S2. NOE columns showing inter-zinc finger interactions between ZF1 and ZF2. *A*, Representative ^{1}H-^{1}H spectral columns from 3D ^{15}N-NOESY spectrum for loop regions. Inter-zinc finger peaks are labeled in red, with intra-zinc fingers in black. *B*, Representative ^{1}H-^{1}H spectral columns from 3D ^{13}C-NOESY spectrum for helix regions. Inter-zinc finger peaks are labeled in red, with intra-zinc fingers in black.

Figure S3. AEBP2 ZF1-3 shows no binding affinity for tested dsDNA and tested G quadruplex. Tzap zinc finger protein was used as a positive control and tested with GC-rich (magenta circle), AT-rich (cyan circle) and T1 (gray circle) dsDNA, respectively. AEBP2 ZF1-3 was tested with GC-rich (red diamond), AT-rich (blue diamond) and T1 (brown diamond) dsDNA and G3A4 (purple diamond), respectively. ND means no detected.

Figure S4. Comparison between TFIIIA zinc fingers (PDB ID: 1TF3) and AEBP2 ZF1-3. *A*, Sequence alignment of AEBP2 zinc fingers. The residues coordinating zinc ions are shown in red and marked with red star. Residues corresponding to DNA binding zinc fingers involved in DNA interaction are labeled with number 6, 3, 2, -1 and marked with black square. *B*, Representation of TFIIIA zinc fingers. Upward is the cartoon view with residues on -1, +2, +3, +6 shown in sticks. Downward is the electrostatic surface with DNA shown in cartoon. color scheme as for Figure 1. *C*, Representation of AEBP2 ZF1-3. Upward is the cartoon view with residues on -1, +2, +3, +6 shown in sticks. Downward is the electrostatic surface potential representation.

Figure S5. Arg380 and Lys382 are crucial for AEBP2 RRK motif bound to RBBP4. *A*, ITC assays for determining the crucial residues between RBBP4 and $AEBP2_{379\text{-}390}$ peptide mutants. Data were fitted to a one-site binding model using Origin 7. *B*, Sequence alignment of AEBP2. Representative sequences of the corresponding FOG1, PHF6, and histone H3 motifs are aligned with $AEBP2_{379\text{-}390}$. The residues crucial for binding affinity are shown in red. *C*, Comparison of $AEBP2_{379\text{-}390}$ peptide binding properties of RBBP4 with other reported complex structures. The FOG-1 (PDB ID: 2XU7), PHF6 (PDB ID: 4R7A), H3 (PDB ID: 2YBA) and AEBP2 are shown as magenta, cyan, blue and yellow, respectively, with relative RBBP4 or Nurf55 shown as violet, light cyan, green and gray, respectively.

Figure S6. Coomassie staining of SDS-PAGE gel for gel-filtration assays corresponding to Figure 2E.

Figure S1.

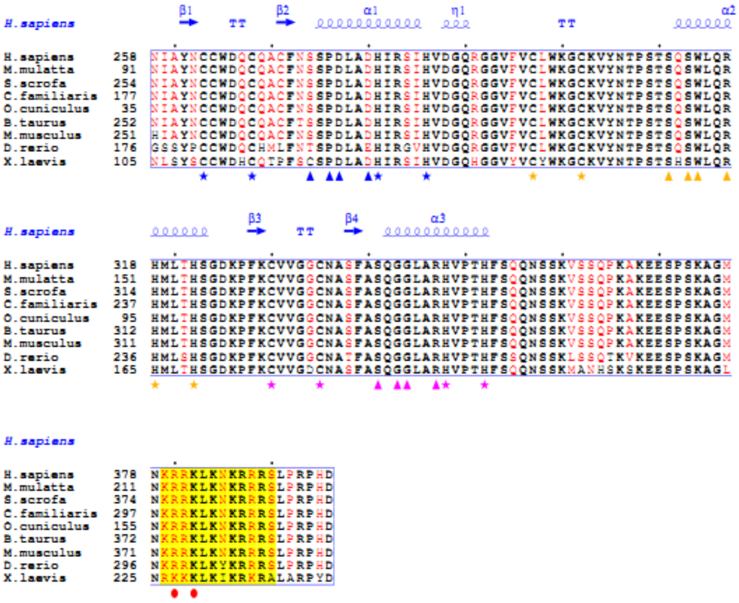
H.sapiens
β1 TT β2 α1 η1 TT α2
H.sapiens 258 NIAYNCCWDQCQACFNSSPDLADHIRSIHVDGQRGGVFVCLWKGCKVYNTPSTSQSWLQR
M.mulatta 91 NIAYNCCWDQCQACFNSSPDLADHIRSIHVDGQRGGVFVCLWKGCKVYNTPSTSQSWLQR
S.scrofa 254 NIAYNCCWDQCQACFNSSPDLADHIRSIHVDGQRGGVFVCLWKGCKVYNTPSTSQSWLQR
C.familiaris 177 NIAYNCCWDQCQACFNSSPDLADHIRSIHVDGQRGGVFVCLWKGCKVYNTPSTSQSWLQR
O.cuniculus 35 NIAYNCCWDQCQACFNSSPDLADHIRSIHVDGQRGGVFVCLWKGCKVYNTPSTSQSWLQR
B.taurus 252 NIAYNCCWDQCQACFTSSPDLADHIRSIHVDGQRGGVFVCLWKGCKVYNTPSTSQSWLQR
M.musculus 251 HIAYNCCWDQCQACFNSSPDLADHIRSIHVDGQRGGVFVCLWKGCKVYNTPSTSQSWLQR
D.rerio 176 GSSYPCCWDQCHMLFNTSPDLAEHIRGVHVDGQRGGVFVCLWKGCKVYNTPSTSQSWLQR
X.laevis 105 NLSYSCCWDHCQTPFSCSPDLADHIRSIHVDGQHGGVYVCYWKGCKVYNTPSTSHSWLQR
H.sapiens
β3 TT β4 α3
H.sapiens 318 HMLTHSGDKPFKCVVGGCNASFASQGGLARHVPTHFSQQNSSKVSSQPKAKEESPSKAGM
M.mulatta 151 HMLTHSGDKPFKCVVGGCNASFASQGGLARHVPTHFSQQNSSKVSSQPKAKEESPSKAGM
S.scrofa 314 HMLTHSGDKPFKCVVGGCNASFASQGGLARHVPTHFSQQNSSKVSSQPKAKEESPSKAGM
C.familiaris 237 HMLTHSGDKPFKCVVGGCNASFASQGGLARHVPTHFSQQNSSKVSSQPKAKEESPSKAGM
O.cuniculus 95 HMLTHSGDKPFKCVVGGCNASFASQGGLARHVPTHFSQQNSSKVSSQPKAKEESPSKAGM
B.taurus 312 HMLTHSGDKPFKCVVGGCNASFASQGGLARHVPTHFSQQNSSKVSSQPKAKEESPSKAGM
M.musculus 311 HMLTHSGDKPFKCVVGGCNASFASQGGLARHVPTHFSQQNSSKVSSQPKAKEESPSKAGM
D.rerio 236 HMLSHSGDKPFKCVVGGCNATFASQGGLARHVPTHFSSQNSSKLSSQTKVKEESPSKAGM
X.laevis 165 HMLTHSGDKPFKCVVGDCNASFASQGGLARHVPTHFSQQNSSKMANHSKSKEESPSKAGL
H.sapiens
H.sapiens 378 NKRRKLKNKRRRSLPRPHD
M.mulatta 211 NKRRKLKNKRRRSLPRPHD
S.scrofa 374 NKRRKLKNKRRRSLPRPHD
C.familiaris 297 NKRRKLKNKRRRSLPRPHD
O.cuniculus 155 NKRRKLKNKRRRSLPRPHD
B.taurus 372 NKRRKLKNKRRRSLPRPHD
M.musculus 371 NKRRKLKNKRRRSLPRPHD
D.rerio 296 NKRRKLKYKRRRSLPRPHD
X.laevis 225 NRKKKLKIKRKRALARPYD

Figure S2.

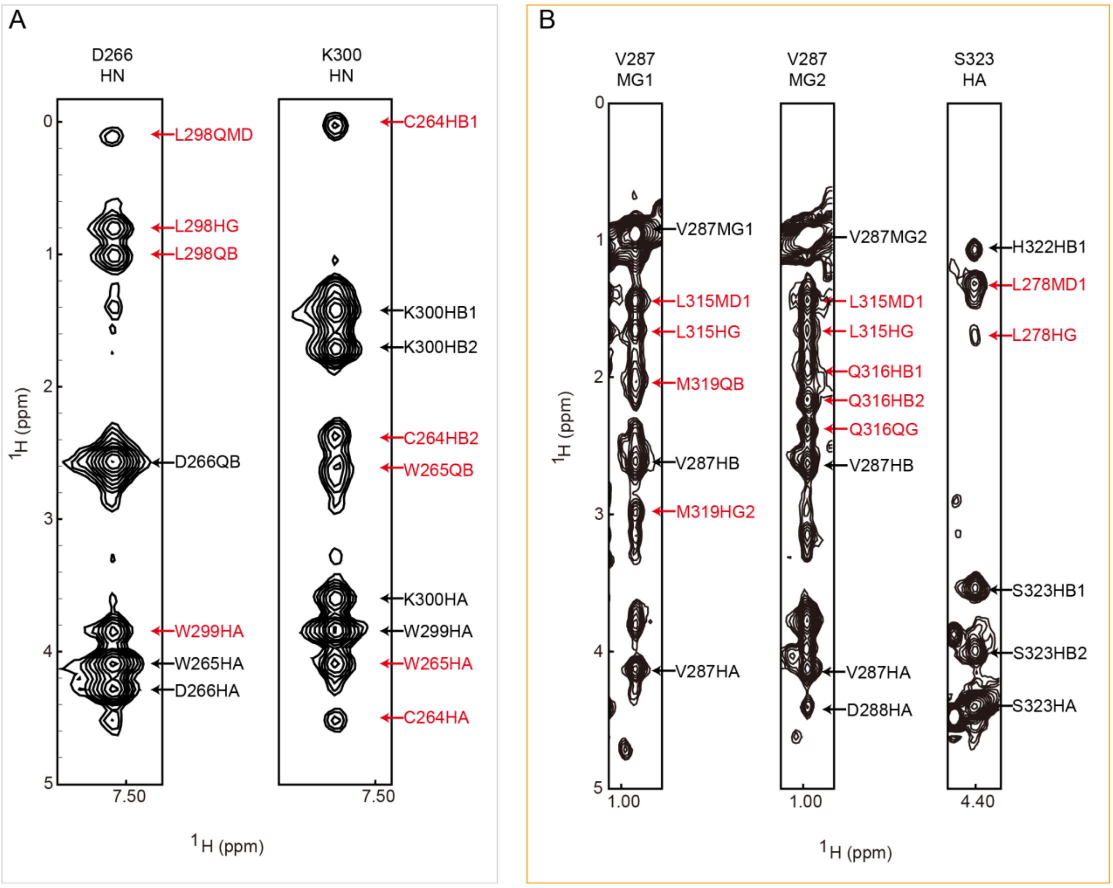
A
D266
HN
K300
HN
L298QMD
L298HG
L298QB
D266QB
W299HA
W265HA
D266HA
C264HB1
K300HB1
K300HB2
C264HB2
W265QB
K300HA
W299HA
W265HA
C264HA
7.50
7.50
1H (ppm)
1H (ppm)
B
V287
MG1
V287
MG2
S323
HA
V287MG1
L315MD1
L315HG
M319QB
V287HB
M319HG2
V287HA
V287MG2
L315MD1
L315HG
Q316HB1
Q316HB2
Q316QG
V287HB
V287HA
D288HA
H322HB1
L278MD1
L278HG
S323HB1
S323HB2
S323HA
1.00
1.00
4.40
1H (ppm)
1H (ppm)

Figure S3.

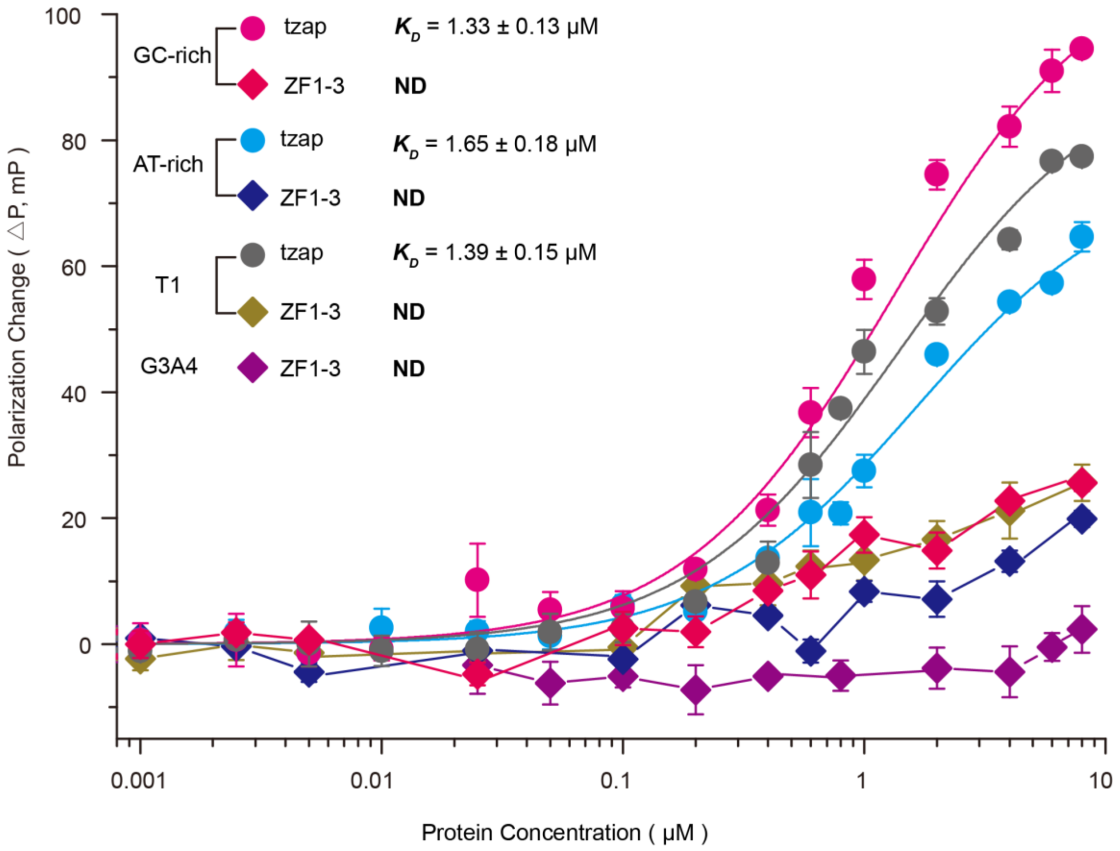
GC-rich
tzap
K_D = 1.33 ± 0.13 μM
ZF1-3
ND
AT-rich
tzap
K_D = 1.65 ± 0.18 μM
ZF1-3
ND
T1
tzap
K_D = 1.39 ± 0.15 μM
ZF1-3
ND
G3A4
ZF1-3
ND
Polarization Change (△P, mP)
100
80
60
40
20
0
0.001
0.01
0.1
1
10
Protein Concentration (μM)

Figure S4.

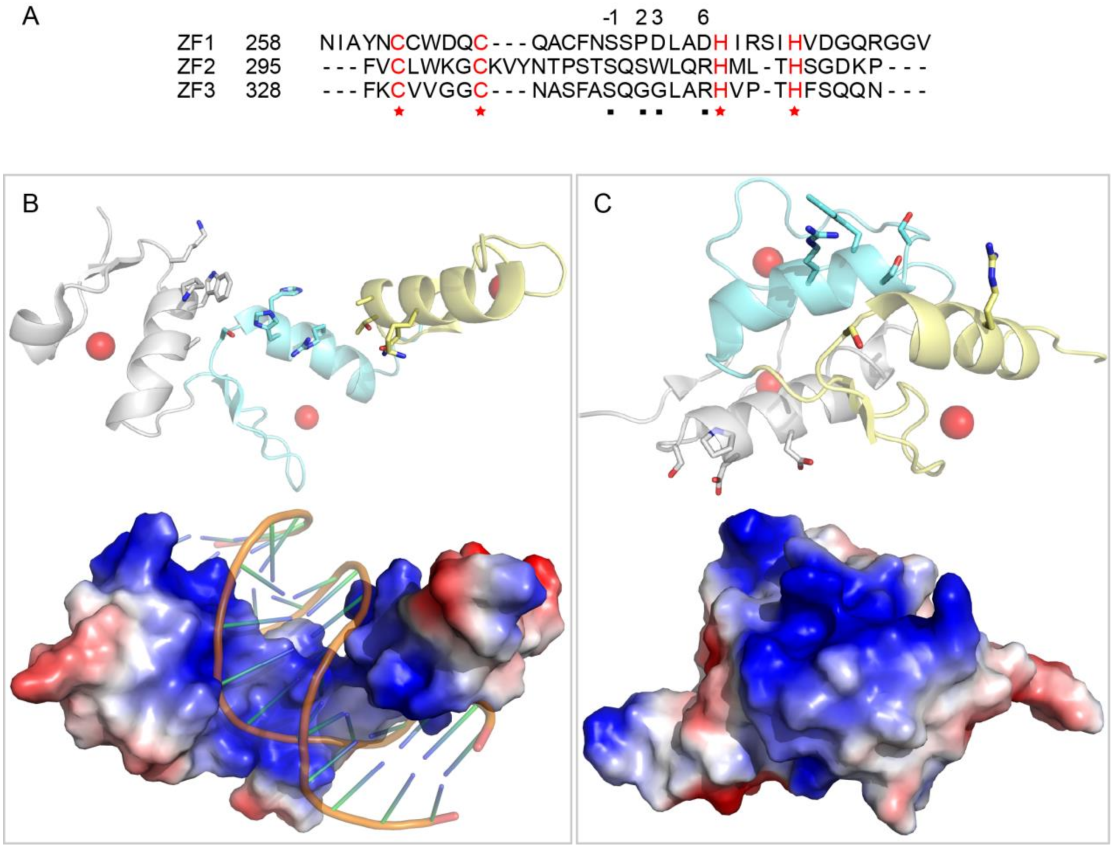
A
-1 2 3 6
ZF1 258 NIAYNCCWDQC---QACFNSSPDLADHIRSIHVDGQRGGV
ZF2 295 ---FVCLWKGCKVYNTPSTSQSWLQRHML-THSGDKP---
ZF3 328 ---FKCVVGGC---NASFASQGGLARHVP-THFSQQN---
B
C

Figure S5.

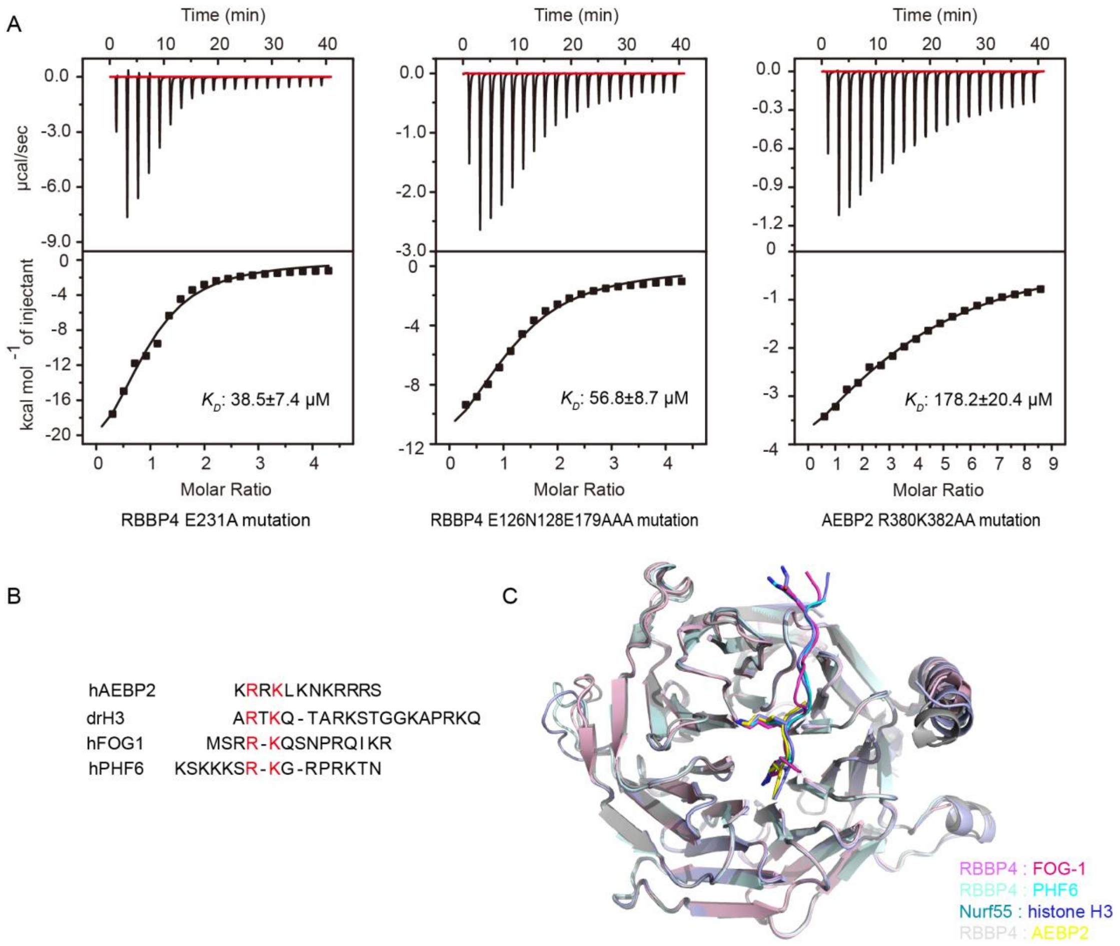
A
Time (min)
μcal/sec
kcal mol-1 of injectant
Molar Ratio
KD: 38.5±7.4 μM
RBBP4 E231A mutation
KD: 56.8±8.7 μM
RBBP4 E126N128E179AAA mutation
KD: 178.2±20.4 μM
AEBP2 R380K382AA mutation
B
hAEBP2 KRRKLKNKRRRS
drH3 ARTKQ-TARKSTGGKAPRKQ
hFOG1 MSRR-KQSNPRQIKR
hPHF6 KSKKKSR-KG-RPRKTN
C
RBBP4 : FOG-1
RBBP4 : PHF6
Nurf55 : histone H3
RBBP4 : AEBP2

Figure S6.

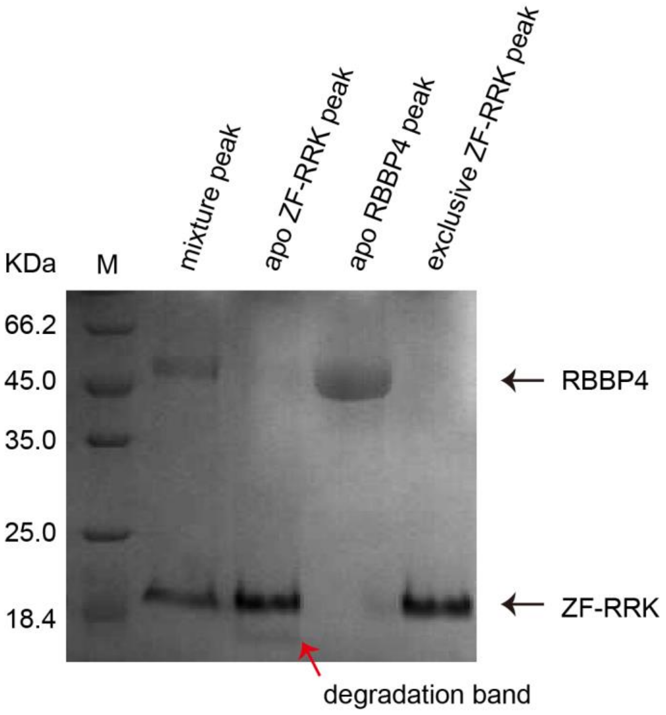
mixture peak
apo ZF-RRK peak
apo RBBP4 peak
exclusive ZF-RRK peak
KDa
M
66.2
45.0
35.0
25.0
18.4
RBBP4
ZF-RRK
degradation band

SUPPLEMENTARY TABLEs

Table S1. Structure calculation statistics of AEBP2 ZF1-3

**Number of restraints (CYANA upper limits)**	
intra-residue [i = j]	353
sequential [	i - j	= 1]	472
medium range [1 <	i - j	< 5]	377
long range [	i - j	≥ 5]	430
Total NOE	1635
Dihedrals	129
Zinc coordination restraints	54
**Mean rmsd from Idealized Covalent Geometry**	
bonds (Å)	0.01204±0.00032
angles (degrees)	1.27459 ± 0.04300
improper (degrees)	1.43641 ±0.09455
**Mean rmsd from Experiment Restraints**	
distance (Å)	0.01985±0.00116
Cdih (degrees)	0.57365±0.12224
**RMSD (Å)**	
all bb / heavy	3.0/3.4
261-352 bb / heavy	0.602/0.954
261-286 bb / heavy	0.352/0.905
295-322 bb / heavy	0.313/0.749
328-352 bb / heavy	0.285/0.741
**Ramachandran (%)***	
Favourable	88.6
Additionall allowed	11.4
Generously	0
Disallowed	0

*Calculated using nonglycine and nonproline residues 261-265, 268-324, 327-353 with the program PROCHECK-NMR to access the quality of the structure.

Table S2. The observed inter-finger NOE between ZF1 and ZF2.

ZF1		ZF2			ZF1		ZF2	
Residue	Atom	Residue	Atom		Residue	Atom	Residue	Atom
W265	HA	W299	HA		D266	QB	L298	HA
W265	HN	W299	HA		D266	QB	L298	HG
W265	HN	W299	HD1		D266	QB	L298	QMG
D266	HA	K300	QD		W265	HA	W299	HA
D266	QB	L298	QMD		W265	HA	W299	HN
D266	HN	L298	QMD		D266	HA	K300	QD
D266	HN	L298	HG		D266	HA	K300	QE
D266	HN	L298	QB		C264	HB1	K300	HN
D266	HN	W299	HA		C264	HB2	K300	HN
L278	MD1	S323	HB2		W265	QB	K300	HN
L278	MD1	S323	HA		W265	HA	K300	HN
A279	HA	H322	HB1		C264	HA	K300	HN
A279	MB	S323	HB1		V287	MG2	L315	MD1
I282	MD1	S323	HB1		V287	HA	L315	MD1
I282	MD2	M319	HG2		V287	QMG	L315	MD2
I282	MG2	M319	HA		V287	QMG	Q316	HA
V287	HA	L315	MD1		V287	HB	Q316	HA
V287	HA	L315	HG		V287	QMG	Q316	HE22
V287	HA	M319	HG2		V287	QMG	Q316	HE21
V287	MG1	L315	MD1		I282	MG2	M319	HA
V287	MG1	L315	HG		V287	MG1	M319	HG2
V287	MG1	M319	QB		V287	MG1	M319	HG1
V287	MG1	M319	HG2		L278	MD1	S323	HA
V287	MG2	L315	MD1		L278	HG	S323	HA
V287	MG2	L315	HG		L278	MD1	S323	HB1
V287	MG2	Q316	HB1		A279	MB	S323	HB1
V287	MG2	Q316	HB2		L278	MD1	S323	HB2
V287	MG2	Q316	QG		A279	MB	S323	HB2

Table S3. Data collection and refinement statistics of AEBP2$_{379\text{-}390}$ bound to RBBP4.

**Data collection**	
Space group	*P* 2_1
Cell dimensions	
a, b, c (Å)	76.76, 59.45, 102.44
α, β, γ (°)	90.00, 94.33, 90.00
Wavelength (Å)	0.9792
Resolution (Å)	36.76 -2.15 (2.23-2.15)
R_{merge} (%)	9.8 (66.1)
$I / \sigma I$	14.11 (2.19)
Completeness (%)	99.9 (100.0)
Redundancy	3.9 (3.7)
**Refinement**	
Resolution (Å)	36.76 -2.15 (2.23-2.15)
No. reflections	50618 (4686)
R_{work} / R_{free} (%)	16.90/21.86
No. of atoms	6590
Protein	6087
Peptide	92
Water	411
B-factors (Å^2)	37.76
Protein	37.06
Peptide	65.94
Water	41.78
R.M.S. deviations	
Bond length (Å)	0.007
Bond angles (°)	0.92
Ramachandran values	
Most favored (%)	97.4
Additional allowed (%)	2.6
Outliers (%)	0

*Values in parentheses are for highest-resolution shell.

MATERIALS AND METHODS

AEBP2 protein preparation.

All clones were amplified by PCR from the human cDNA library and ligased into p28 or pGEX plasmid. Proteins were expressed in *Escherichia coli* BL21(DE3)-Gold (Stratagene) at 16 ℃ for 24 hours. All proteins were prepared from cells growing in LR medium supplemented with 0.1 mM $ZnSO_4$, $^{15}NH_4Cl$, with or without $^{13}C_6$ -glucose. The proteins were purified using the Ni-chelating Sepharose™ Fast Flow column (GE Healthcare) and then further purified by chromatography on a HiLoad Superdex 200 16/60 column (GE Healthcare).

Nuclear magnetic resonance spectroscopy.

All NMR spectra were collected at 298K on a Bruker DMX600 spectrometer equipped with a cryoprobe. The protein was concentrated to 0.6-0.8 mM in buffer consisting of 25 mM NaH_2PO_4 (pH 6.3), 100 mM NaCl, and 5 mM DTT with 10% D_2O. Sequential assignment were conducted from CBCANH, CBCA(CO)NH, CC(CO)NH-TOCSY, HCC(CO)NH-TOCSY in H_2O and HCCH-COSY, HCCH-TOCSY in D_2O. NOE distance restraints were obtained from 2D 1H-1H NOESY, 3D ^{15}N-edited and ^{13}C-edited NOESY. All spectra were processed using the suite of programs provided in the NMRPipe/NMRDraw software package (Delaglio et al., 1995) and analyzed in Sparky (provided by T. D. Goddard and D. G. Kneller, University of California, San Francisco).

NMR structure calculation

Structure calculation of all three AEBP2 zinc fingers was performed in CYANA using fully automated NOESY assignment (Guntert, 2003). Distance restraints were generated and calibrated automatically by CYANA using 2D 1H-1H NOESY, ^{15}N- and ^{13}C-edited NOESY-HSQC peak lists. The restraints for backbone dihedral angles were determined using TALOS+ (Shen et al., 2009). Additional restraints were introduced for distances between the zinc ion and zinc-coordinating atoms to enforce tetrahedral geometry of the zinc center. The structure was recalculated by CNS software (Brunger et al., 1998) and 20 lowest-energy conformers were selected. The quality of the ensemble of 20 lowest energy structures was assessed using PROCHECK-NMR (Laskowski et al., 1996) and Protein Structure Validation Server (PSVS) (Bhattacharya et al., 2007). NMR calculation and refinement data are displayed in Table 1. The structure figures were prepared with MOLMOL (Koradi et al., 1996) and PyMOL (DeLano, 2002) (DeLano Scientific San Carlos, California USA).

Fluorescence Polarization Assays (FPA)

The dsDNA segments used for fluorescence polarization assays included T1 (5' GATCTTTAGTTAACTTCCTCTCTGTCTGCAGGCCACTCCAGATC 3'), AT-rich (5' ATAATTTATATTTATTATTTTATTATAATTTATATAATTTATAT 3'), GC-rich (5' GCGGCCCGCGCCCGCCGCCCCGCCGCGGCCCGCGCGGCCCGCGC 3'), and G3A4 (5' AAAAAAGGGAAAAGGGAAAAGGGAAAAGGGAAAAAAAAAA 3') and were synthesized with the sense chain labeled (5'-FAM) (Carboxyfluorescein) by

Takara Bio Inc. (Dalian, China). Two stands of complementary 44-nt single-stranded DNA were annealed to form dsDNA: T1, GC-rich, and AT-rich, respectively. The assays of T1, GC-rich and AT-rich were performed in buffer (25 mM Tris, pH 7.4, 150 mM NaCl) at 20 ℃ using a SpectraMax M5 Microplate Reader system (Molecular Devices). G3A4 was dissolved and the assay of G3A4 was performed in buffer (25mM Tris, pH 7.4, 150mM KCl) for the formation of G quadruplex. The wavelengths of fluorescence excitation and emission were 490 nM and 525 nM, respectively. Each 96-well plate contained 50 nM of fluorescently labeled (5' -FAM) DNA probe and different amounts of AEBP2 ZF1-3 with a final volume of 200 μl. The data were further analyzed using Origin9, as previously described (Liu et al., 2014).

RBBP4 protein preparation.

The gene encoding full-length human RBBP4 (residues 1–425; UniProt accession number Q09028) was cloned between the EcoR I and Xba I sites of a pFastBacTM HT B vector. The pFastBacTM HT B construct was expressed in High Five insect cells infected with baculovirus utilizing the Bac-to-Bac system (Invitrogen) according to the manufacturer’s instructions. The cells were harvested and resuspended in lysis buffer (25 mM Tris, 500 mM NaCl, pH 8.0). Cells were lysed by sonication and clarified by ultracentrifugation. The protein was purified with Ni-chelating SepharoseTM Fast Flow column (GE Healthcare) and further purified by chromatography on a HiLoad Superdex 200 16/60 column (GE Healthcare). The

protein was then concentrated up to 10 mg/ml in buffer consisting of 2 5 mM Tris (pH 7.4), 150 mM NaCl for further experiments.

AEBP2 peptide synthesis.

Wild-type AEBP2 (residues 379-390) and mutant peptides used here were chemically synthesized and purified (to 95% purity) by GL Biochem (Shanghai, China).

Isothermal Titration Calorimetry (ITC) Experiments.

ITC measurements were carried out at 25 ℃ using a MicroCal iTC200 titration calorimeter (GE Healthcare). RBBP4 and AEBP2 peptides were dissolved separately in ITC buffer (25 mM Tris, pH 7.4, 150 mM NaCl). The AEBP2 peptides were injected into RBBP4 at time intervals of two minutes between each 2 μl injection (20 injections in total). The data were analyzed using one-site binding model via the MicroCal Origin 7.0 software package (provided by the manufacturer).

Crystallization and Structure Determination.

Concentrated RBBP4 protein (10 mg/ml) was incubated overnight at 4 ℃ with $AEBP2_{379-390}$ peptide at a molar ratio of 1:2 . Additionally, 1 μl RBBP4-AEBP2 complex drops were mixed with 1 μl of crystallization solution using the sitting drop vapor diffusion method. Single crystals were obtained at 8-12 ℃ for a week in 0.2 M Ammonium sulfate; 0.1 M MES monohydrate pH 6.5; 30% w/v Polyethylene glycol monomethyl ether 5,000 (HAMPTOM RESEARCH). Crystals were harvested,

soaked in mother liquor supplemented with 30% glycerol and then flash frozen in liquid nitrogen. X-ray diffraction data were collected on beamline 17U1 at the Shanghai Synchrotron Radiation Facility (SSRF). The initial data were processed using HKL2000 (Battye et al., 2011). The structure was then solved by molecular replacement using RBBP4 (PDB ID: 3GFC) (Xu and Min, 2011) as a search model via MOLREP (Vagin and Teplyakov, 2010). The model was further built and refined using Coot (Emsley et al., 2010), Refmac (Murshudov et al., 2011) and Phenix (Afonine et al., 2012). Crystal diffraction data and refinement statistics are displayed in Table 2.

Gel Filtration Assay

RBBP4 and AEBP2 ZF-RRK were concentrated in buffer (25 mM Tris, pH 7.4, 150 mM NaCl) and mixed with molar ratio of 1 : 2.5. The mixture, apo RBBP4, apo AEBP2 ZF-RRK were conducted on a same Superdex™ 200 10/300 GL column, respectively.

Coordinates

Coordinates, chemical shifts and peaks of AEBP2 three zinc fingers have been deposited in the Protein Data Bank (PDB) under the accession code 5Y0U, corresponding to the Biological Magnetic Resonance Bank (BMRB) under the accession code 36108. Coordinates and structure factors of RBBP4 bound to the $AEBP2_{379\text{-}390}$ peptide complex have been deposited in PDB under the accession code

5Y1U.

REFERENCES

Afonine, P.V., Grosse-Kunstleve, R.W., Echols, N., Headd, J.J., Moriarty, N.W., Mustyakimov, M., Terwilliger, T.C., Urzhumtsev, A., Zwart, P.H., and Adams, P.D. (2012). Towards automated crystallographic structure refinement with phenix.refine. Acta crystallographica Section D, Biological crystallography 68, 352-367.

Battye, T.G., Kontogiannis, L., Johnson, O., Powell, H.R., and Leslie, A.G. (2011). iMOSFLM: a new graphical interface for diffraction-image processing with MOSFLM. Acta crystallographica Section D, Biological crystallography 67, 271-281.

Bhattacharya, A., Tejero, R., and Montelione, G.T. (2007). Evaluating protein structures determined by structural genomics consortia. Proteins 66, 778-795.

Brunger, A.T., Adams, P.D., Clore, G.M., DeLano, W.L., Gros, P., Grosse-Kunstleve, R.W., Jiang, J.S., Kuszewski, J., Nilges, M., Pannu, N.S., *et al.* (1998). Crystallography & NMR system: A new software suite for macromolecular structure determination. Acta crystallographica Section D, Biological crystallography 54, 905-921.

Delaglio, F., Grzesiek, S., Vuister, G.W., Zhu, G., Pfeifer, J., and Bax, A. (1995). Nmrpipe - a Multidimensional Spectral Processing System Based on Unix Pipes. Journal Of Biomolecular Nmr 6, 277-293.

DeLano, W.L. (2002). PyMOL: An Open-Source Molecular Graphics Tool. DeLano Scientific San Carlos, California USA.

Emsley, P., Lohkamp, B., Scott, W.G., and Cowtan, K. (2010). Features and development of Coot. Acta crystallographica Section D, Biological crystallography 66, 486-501.

Guntert, P. (2003). Automated NMR protein structure calculation. Progress In Nuclear Magnetic Resonance Spectroscopy 43, 105-125.

Koradi, R., Billeter, M., and Wuthrich, K. (1996). MOLMOL: a program for display and analysis of macromolecular structures. Journal of molecular graphics 14, 51-55, 29-32.

Laskowski, R.A., Rullmannn, J.A., MacArthur, M.W., Kaptein, R., and Thornton, J.M. (1996). AQUA and PROCHECK-NMR: programs for checking the quality of protein structures solved by NMR. J Biomol NMR 8, 477-486.

Liu, Z., Li, F., Ruan, K., Zhang, J., Mei, Y., Wu, J., and Shi, Y. (2014). Structural and functional insights into the human Borjeson-Forssman-Lehmann syndrome-associated protein PHF6. The Journal of biological chemistry 289, 10069-10083.

Murshudov, G.N., Skubak, P., Lebedev, A.A., Pannu, N.S., Steiner, R.A., Nicholls, R.A., Winn, M.D., Long, F., and Vagin, A.A. (2011). REFMAC5 for the refinement of macromolecular crystal structures. Acta crystallographica Section D, Biological crystallography 67, 355-367.

Shen, Y., Delaglio, F., Cornilescu, G., and Bax, A. (2009). TALOS+: a hybrid method for predicting protein backbone torsion angles from NMR chemical shifts. J Biomol NMR 44, 213-223.

Vagin, A., and Teplyakov, A. (2010). Molecular replacement with MOLREP. Acta

crystallographica Section D, Biological crystallography 66, 22-25.

Xu, C., and Min, J. (2011). Structure and function of WD40 domain proteins. Protein & cell 2, 202-214.
